# Heaviness-brightness correspondence and stimulus-response compatibility

**DOI:** 10.3758/s13414-019-01963-6

**Published:** 2020-01-02

**Authors:** Peter Walker, Gabrielle Scallon, Brian J Francis

**Affiliations:** 1 Department of Psychology, Lancaster University, Lancaster, LA1 4YF UK; 2 Provost’s Office, Sunway University, Bandar Sunway, Subang Jaya, Selangor Malaysia; 3 Department of Mathematics and Statistics, Lancaster University, Lancaster, UK

**Keywords:** Cross-sensory correspondences, Heaviness, Brightness, Stimulus-response compatibility

## Abstract

Cross-sensory correspondences can reflect crosstalk between aligned conceptual feature dimensions, though uncertainty remains regarding the identities of all the dimensions involved. It is unclear, for example, if heaviness contributes to correspondences separately from size. Taking steps to dissociate variations in heaviness from variations in size, the question was asked if a heaviness-brightness correspondence will induce a congruity effect during the speeded brightness classification of simple visual stimuli. Participants classified the stimuli according to whether they were brighter or darker than the mid-gray background against which they appeared. They registered their speeded decisions by manipulating (e.g., tapping) the object they were holding in either their left or right hand (e.g., left for *bright*, right for *dark*). With these two otherwise identical objects contrasting in their weight, stimuli were classified more quickly when the relative heaviness of the object needing to be manipulated corresponded with the brightness of the stimulus being classified (e.g., the heavier object for a darker stimulus). This novel congruity effect, in the guise of a stimulus-response (S-R) compatibility effect, was induced when heaviness was isolated as an enduring feature of the object needing to be manipulated. It was also undiminished when participants completed a concurrent verbal memory load task, countering claims that the heaviness-brightness correspondence is verbally mediated. Heaviness, alongside size, appears to contribute to cross-sensory correspondences in its own right and in a manner confirming the far-reaching influence of correspondences, extending here to the fluency with which people communicate simple ideas by manipulating a hand-held object.

## Introduction

In everyday life, people comment on the darkness, heaviness, and roundness of a sound, the sharpness of a taste, and the darkness, heaviness, and thickness of a perfume.^1^ With such features normally being linked to a different modality than the one to which they are being applied here, it seems that stimuli encoded in different sensory domains can share some of the same qualities (see Deroy, Crisinel, & Spence, 2013, for a review relating to odor).

Brightness,^2^ heaviness, sharpness, and thickness are feature dimensions that appear to be aligned with each other in a specific way, with stimuli encoded in different domains able to share their relative positioning on the dimensions (see Walker & Walker, 2016). The systematic manner in which progressively more extreme values on one dimension (e.g., the progressively higher pitch of a sound) map onto progressively more extreme values on another dimension (e.g., the progressively brighter appearance of a visual stimulus) is what the term cross-sensory *correspondence,* rather than mere *association,* is intended to capture.

At the most abstract level, though not necessarily at all levels, correspondences are thought to involve dimensions that are modality independent and conceptual in nature^3^ (see Karwoski, Odbert, & Osgood, 1942; Martino & Marks, 1999, 2001; Melara & Marks, 1990; Velasco, Woods, Marks, Cheok, & Spence, 2016; Walker & Smith, 1984; Walker, Walker, & Francis, 2015). For the correspondence between auditory pitch and visual brightness, for example, it would be the modality-independent concepts of elevation and brightness that are aligned.^4^ This means that any domain-specific features feeding into these concepts are able to engage with the elevation-brightness correspondence.

Much of the evidence concerning cross-sensory correspondences has come from examining how contrasting values of auditory pitch map onto a range of visual features, most notably size, brightness, pointiness, and visuo-spatial elevation (see Spence, 2011, and Walker, 2016a, for reviews). However, if the dimensions underlying correspondences are modality independent and conceptual in nature, the same correspondences should appear whichever sensory channel is engaged to probe them. That a single network of interconnected feature dimensions can be responsible for cross-sensory correspondences is what is suggested by the cross-sensory metaphors used in language (see Marks, 1978), and there is other evidence to confirm this. For example, it is not just smaller visual objects that are judged to be brighter, faster, higher in space, sharper, thinner, and to make higher pitch sounds than bigger objects, smaller objects explored by touch alone are also judged to have these cross-sensory features (Walker & Walker, 2012; Walker, Walker, & Francis, 2012) (see Ludwig & Simner, 2013, for further evidence that object features encoded through touch, such as pointiness, can enter into correspondence with visual features, including brightness and color saturation). Similarly, not only does the up and down vertical motion of a visual object correspond with the rising and falling pitch of a sound, so too does the vertical motion of a brush stroke on a person's back (Nava, Grassi, & Turati, 2016). By the same token, it follows that the felt heaviness of an object should also contribute to the correspondences that are evident when stimuli in other domains are varying systematically in their judged heaviness. For example, with darker visual objects being judged to be heavier in weight than brighter visual objects, unseen objects that feel relatively heavy when lifted should be judged to be darker than comparable objects that feel less heavy. The rationale for the present study begins with this presumed involvement of felt heaviness in cross-sensory correspondences. First, however, it is important to evaluate the existing evidence for its involvement.

## Heaviness as a feature in cross-sensory correspondences

Heaviness is a feature consistently implicated in cross-sensory correspondences: Bigger visual objects are judged to be heavier than otherwise identical but smaller visual objects (Walker & Smith, 1985; Walker et al., 2012), and lower pitch sounds are judged to be heavier than higher pitch sounds (Walker & Smith, 1984; Walker et al., 2012), as are curved geometric shapes compared to their size-matched pointy equivalents (Walker et al., 2012). Furthermore, on the basis of vision alone, darker colored balls are judged to be heavier than otherwise identical brighter balls (Walker, Francis, & Walker, 2010; Walker, 2012b).

Encouraging as it is, such evidence for the involvement of heaviness in cross-sensory correspondences is limited in three respects. First, heaviness is a feature induced by some other cross-sensory feature contrast, rather than the feature that is itself inducing a cross-sensory feature association. Second, heaviness is represented verbally, rather than as the felt heaviness of a tangible object. Third, because the same cross-sensory features associated with heaviness are also associated with size (e.g., bigger, like heavier, is also both darker and lower in pitch), the assumed involvement of heaviness in cross-sensory correspondences could be secondary to the involvement of size: Lower pitch sounds and curved shapes could appear to be heavier only to the extent that they seem to be bigger.

There are grounds to caution against dismissing the involvement of heaviness as being illusory in this way. For example, when curved and pointy visual shapes are carefully matched for their perceived size, and then rated for their cross-sensory features, curvedness aligns itself with heaviness despite the absence of a difference in perceived size (Walker et al., 2012). Similarly, darker colored objects are expected to feel heavier than equivalent but lighter colored objects (Walker, Francis, & Walker, 2010), even though lighter colored objects are likely to be perceived as being bigger than darker objects (Gundlach & Macoubray, 1931; Robinson, 1954; Wallis, 1935). Walker et al.'s (Walker, Francis, & Walker, 2010) observation provides part of the motivation for the present attempt to demonstrate that a functionally bi-directional heaviness-brightness correspondence exists, distinct from a size-brightness correspondence.

Of particular interest for the present study, Walker, Scallon, and Frances (2016) took steps to isolate the effects of felt heaviness from those of size. They created a set of nine solid cylindrical objects in which size and weight were varied independently by crossing three values of weight against three values of size. With the objects remaining hidden from view, participants lifted them by hand and conveyed a judgment as to their assumed brightness. Objects that felt lighter in weight were judged to be brighter, with these judgements of brightness tracking the changes in felt heaviness that were induced by the size-weight illusion. Moreover, it was the perceived heaviness of an object, rather than its size, that dominated in determining an object's cross-sensory features (though felt heaviness was itself determined by the size of an object in combination with its weight, as per the size-weight illusion).

## Speeded classification

Explicit judgments regarding an item's cross-sensory features do not provide the most persuasive evidence for correspondences. Such judgments are limited in two ways. First, because the explicit judgment procedure is relatively transparent regarding what the experimenter is wishing to probe, it is vulnerable to claims regarding demand characteristics. Second, what are meant to be judgments reflecting associations among perceptual features might also reflect, to some extent at least, purely verbal associations, and on occasion the judgments might be explained solely on the basis of such associations. To illustrate, the rating scales on which individual feature values are judged are typically defined for participants with verbal labels. And, regarding the stimulus feature being judged, even when this is not itself presented verbally, there is nothing in place to discourage participants from verbally recoding it. Hence, any pre-existing associations between the name of the feature being judged, and the names of the cross-sensory features defining the rating scale, have the opportunity to influence the judgement.

More persuasive evidence serving to identify cross-sensory correspondences comes from speeded classification tasks, where participants classify a feature in one sensory domain in the presence of a task-irrelevant feature in another sensory domain (e.g., they classify a visual shape as pointy or curved when it is accompanied by a task-irrelevant sound that is either high-pitched or low-pitched). Observing a congruity effect, wherein classification decisions are made more quickly and/or more accurately when the target feature is accompanied by an incidental stimulus possessing a corresponding feature (e.g., a pointy shape accompanied by a high-pitch sound), helps to confirm the existence of a correspondence between the two feature dimensions. Given that instructions for the speeded classification task need not, and normally do not, make any explicit reference to the task-incidental stimuli, along with the obvious fact that these stimuli are task-incidental, makes it unlikely that participants deliberately acknowledge their features, let alone verbally recode them.

Congruity effects in speeded classification have confirmed the same cross-sensory correspondences observed when participants make explicit judgements about the cross-sensory features of stimuli (e.g., Chiou & Rich, 2012; Evans & Treisman, 2010; Gallace & Spence, 2006; Occelli, Spence, & Zampini, 2009; Ro, Hsu, Yasar, Elmore, & Beauchamp, 2009; Walker, 2012a). For example, performance in a brightness-classification task confirms the existence of a brightness-pitch correspondence, with people responding more quickly when, for example, a bright visual stimulus is accompanied by a high-pitch sound (a “brighter” sound) rather than a low-pitch sound (a “darker” sound) (Marks, 1987; Martino & Marks, 1999; Melara, 1989). Such evidence is especially persuasive because the incidental nature of one of the two stimuli means that participants are unlikely to think either they should compare the two stimuli or they should verbally recode them. It is in this way that speeded classification tasks avoid the two limitations associated with explicit judgments of cross-sensory features.

Correspondence-induced congruity effects have also been observed in speeded classification tasks in which the task-irrelevant feature has been a feature of the response by which participants communicate their classification decision. For example, Lidji, Kolinsky, Lochy, and Morais (2007) and Rusconi, Kwan, Giordano, Umilta, and Butterworth (2006) arranged for the two alternative response keys being used in an auditory pitch classification task to be arranged spatially one above the other. They found that participants were able to respond more quickly when the elevation of the response key needing to be pressed was congruent with the pitch of the sound being classified (e.g., a more elevated key for a higher pitch sound). In addition, Walker and Smith (1985) arranged for the two alternative response keys being used in an antonym classification task to contrast in their size, and found that participants responded more accurately and/or more quickly when the size of the response key needing to be pressed was congruent with the antonym being classified (e.g., a larger key, rather than a smaller key, for the antonyms *“strong,” “heavy,” “down,” “bottom”)*.

Finally, in a study that is directly relevant to the work being reported here, Walker and Walker (2012) manipulated a response feature while providing evidence for a size-brightness correspondence in the context of the brightness-classification task. They presented participants with one of six possible solid circles, differing in brightness, on a computer screen. Three of the circles were brighter than the mid-gray background on which all the circles appeared, and three were darker than this background. Participants were required to decide, as quickly as possible, by pressing either the left or right of two response keys, whether each circle was brighter or darker than the background. The two response keys comprised a small (2.5 cm diameter) and a large (7.5 cm diameter) wooden ball, each mounted on a microswitch. In a preliminary study, where participants explored the balls by hand while they remained hidden from view, it was confirmed that the balls had contrasting cross-sensory features, including that the smaller one was judged to be brighter than the bigger one. In the speeded classification task, the response keys were never seen by participants, and the difference in their size was task irrelevant and never referred to by the experimenter. In the interval between successive blocks of trials, and while participants were distracted by engaging with a filler task, the keys were surreptitiously switched to opposite hands, though the same brightness-hand mapping was maintained. Walker and Walker observed the predicted size-brightness congruity effect, with participants responding relatively more quickly when the size of the key needing to be pressed on a trial corresponded with the brightness of the visual stimulus being classified (e.g., the smaller key to confirm that a circle was relatively bright). Because the response keys were fixed in place, and so could not be lifted, Walker and Walker assumed that it was the size of the balls, rather than their heaviness, that was functionally significant. It is possible, however, that participants inferred that the larger key was heavier than the smaller key, and that with this inference the concept of heaviness became the functionally significant feature. That this is feasible is evident from Walker and Smith's (1985) study (see above), where participants classified the words *heavy* and *light* more quickly when doing so required them to press a response key having a corresponding size (i.e., big and small for *heavy* and *light*, respectively).

## The present study

The heaviness-brightness correspondence was examined to address three fundamental issues regarding cross-sensory correspondences. The first issue concerns the identities of the feature dimensions involved in correspondences. As it stands, some of their identities are unclear. For example, sharpness and thinness are strongly linked features, and it remains to be determined if they are each separately involved in correspondences, or if they should be thought of as a single feature dimension. Size and heaviness likewise are sufficiently strongly linked for it to be uncertain if they each contribute separately to cross-sensory correspondences, or if they also should be collapsed into a single feature dimension. Developing a comprehensive account of correspondences requires all the separate feature dimensions needing to be taken into account to be identified.

The second issue concerns the features of behavioral responses to stimuli (such as the key presses typically used as classification responses), and whether these also engage with cross-sensory correspondences. We already know that the relative elevation and size of the keys needing to be pressed are two such features (Lidji et al., 2007; Rusconi et al., 2006; Walker & Smith, 1985; Walker & Walker, 2016). In the present study, it is asked if the heaviness of an object needing to be manipulated to communicate a simple classification decision is also involved in correspondences.

The third issue concerns a practical implication of cross-sensory correspondences. If cross-sensory correspondences can involve features of the behavioral responses to stimuli, it then becomes important to consider how simple ideas are communicated, whether to another person or to a machine. An instance of the former would include the role of correspondences in shaping the prosody adopted to make the spoken communication of simple ideas most effective (see Walker, 2016b, for illustrative examples). An instance of the latter would include how human-machine interfaces might be designed for the effective communication of simple ideas, which in turn would entail arranging for each of the objects needing to be manipulated to convey contrasting brightness classification decisions to have corresponding weight.

With these broad aims in mind, the three experiments reported below make use of the speeded brightness-classification task to secure evidence of the predicted heaviness-brightness correspondence. Steps are taken to dissociate this correspondence from the known size-brightness correspondence, and to isolate the felt heaviness of a response object as the feature inducing a correspondence-based congruity effect. Participants register their brightness classification of visual stimuli by manipulating one of two objects they are holding in their hands (e.g., tapping the *left* object for *bright*, and the *right* object for *dark*), with the two objects being identical except for a task-irrelevant difference in their felt heaviness. The presumed heaviness-brightness correspondence predicts that the visual stimuli will be classified more quickly and/or accurately when the heaviness of the object needing to be manipulated to register the classification decision is congruent with the brightness of the stimulus being classified (e.g., the *heavier* object for a *darker* stimulus). From another important perspective, the present experiments are also designed to demonstrate a novel type of stimulus-response (S-R) compatibility effect, for which the level of compatibility is determined by the presumed cross-sensory correspondence between heaviness and brightness. The final experiment aims to confirm these effects when it is heaviness as an enduring feature of an object needing to be manipulated that is isolated as the task-irrelevant feature (rather than heaviness as a factor linked specifically to the action needing to be taken with the object). Additionally, by examining the effect of a concurrent verbal memory load task on brightness classification, this experiment also assesses if purely verbal associations linking heaviness to brightness are responsible for any correspondence-induced heaviness-brightness congruity effect being observed.

## General method

### Apparatus

#### Stimuli for classification

The experiments were conducted with PsyScript 2.0 running on a dual 2 GHz, PowerMac G5 with a 20-in. computer screen (Apple A1038, 1,680 × 1,050 cinema back-lit LCD display with a refresh rate of 60 Hz). The visual stimuli were a subset of those used by Walker and Walker (2012) in their study of the size-brightness correspondence. The subset comprised four solid circles (4.5 cm diameter) differing in brightness from black to white (2, 42, 150, and 340 cd/m^2^). They were presented in the center of the screen on a mid-gray background (90 cd/m^2^).

#### Response keys

**Fig. 1.**
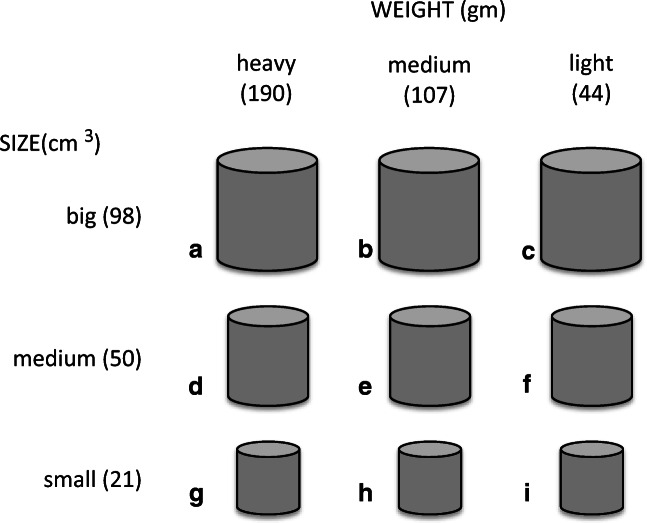
Illustration of the size and weight of each object in the set of nine objects created by crossing weight against size. The objects are labelled to facilitate discussion here, but were not labelled in the experiment. The three alternative values for weight were chosen on the basis that they are a set of values that could arise when objects at the three different sizes are made from the same material (i.e., the same density). Therefore, objects A, E, and I are very close to forming a natural set of objects whose weights indicate they are formed from the same material. From Walker, Scallon, and Francis (2016)

The objects serving as response keys were part of a larger set of nine solid cylindrical objects created for a related study (see Walker et al., 2016). The size and weight of the objects were varied orthogonally, with three values of weight being crossed with three values of size (see Fig. 1). The objects were formed from thin-walled (approx. 1 mm) aluminium tubing filled with evenly distributed fragments of lead mixed in builder’s expanding foam. The ends of the cylinders were smoothed with a fine layer of epoxy resin, after which the cylinders were painted matt gray. Three values for cylinder size (i.e., diameters of 3, 4, and 5 cm, and heights matching these diameters) were crossed with three values for weight (i.e., 44, 107, and 190 g) (i.e., for each size of object three versions differing in weight were created). The weights of the cylinders were manipulated by varying the proportion of lead and builder's foam with which they were evenly filled. The targeted values for weight were chosen on the basis that they should be a set of weights that objects at these three sizes could have when created from the same material (i.e., they reflect the natural co-variation of weight and size). The actual weights achieved were close approximations to the targeted values of 42, 101, and 196 g, where these values correspond to a fixed density of 2 g/cm^3^ (see Fig. 1).

Resting their elbows on the table in front of them, participants held two of the objects, one in each hand, and indicated their classification decision by manipulating the object in whichever hand was appropriate for the classification decision to be made (e.g., left hand for *dark*, right hand for *bright*). In Experiments 1 and 2 they had to tap the appropriate object against a contact-sensitive surface positioned directly beneath their hand. The two surfaces were horizontal circular metal disks (4 cm in diameter) mounted in shallow wooden frames (1.0 cm in depth). The shallowness of the frames allowed participants to hold the objects a short distance (1.5 cm) above the disks until a response needed to be made. The contact-sensitive surfaces were interfaced with the computer, allowing the nature of each classification decision, and the speed with which it was made, to be recorded. The surfaces were also covered with a layer of felt material to dampen any impact sound created when an object made contact with a disk. To further ensure any such sound was not heard, participants wore sound-cancelling headphones through which they listened to a soundtrack comprising random computer keyboard strokes varying unsystematically in their timing. This unpredictable variability in their timing mitigated against participants synchronizing their responses to them. Throughout the experiment, a participant’s hands, the objects they were holding, and the contact-sensitive surfaces against which the objects were tapped, were all hidden from view beneath a thick black cloth.

In Experiment 3, participants manipulated the objects in a different way to indicate their classification decision. A small microswitch button (6 mm diameter, 4 mm deep, and with a 2-mm gap needing to be closed) was fixed to the center of one end of each object. Participants held the two objects in a way that allowed them to close the switch on either object with the thumb of the holding hand. The microswitches were interfaced with the computer, allowing the nature of each classification decision, and the speed with which it was made, to be recorded. Once again, throughout the experiment, participants' hands and the objects they were holding were hidden from view beneath a thick black cloth.

### Experiment 1: Tapping with response keys contrasting in weight and size

Because this was the first experiment in which alternative response keys were to be held in each participant’s hands, and tapped against a surface to register a classification decision, it was decided to allow both weight and size to induce a congruity effect. For this reason, the two objects selected to serve as response keys differed in both weight and size in accordance with the natural covariation of these features when objects are formed from the same material. Specifically, the heaviest + largest and lightest + smallest objects from the set of nine served as the two response keys (i.e., objects a & i in Fig. 1). It was predicted that the same congruity effect reported by Walker and Walker (2012) would again be observed despite the introduction of a difference in weight alongside the difference in size (e.g., faster responses when the heavier + larger object, rather than the lighter + smaller object, was used to classify a stimulus as *dark*). This was because all the evidence points to size and heaviness sharing their cross-sensory correspondences, including that *bigger* and *heavier* both align themselves with *darker*. Once the feasibility of using the procedure had been confirmed with this observation, additional experiments were to be undertaken to dissociate the effects attributable to a difference in heaviness from those attributable to a difference in size.

## Method

### Participants

All of the Lancaster University students participating in the present study were recruited using the Sona Systems experiment management system, with no requirements specified regarding gender, handedness, or first language. Twenty-nine students (18 females and 11 males) aged between 18 and 27 years (mean age = 21.2 years) volunteered to participate in Experiment 1 in exchange for payment or course credits. All except four of the participants were right-handed by self-report, and for 18 participants English was their first language. The first languages of the remaining participants were Bulgarian (*n* = 2), Chinese/Cantonese/Mandarin (*n* = 4), Greek (*n* = 1), Hindi (*n* = 3), and Malay (*n* = 1). Ethical approval for this and the other experiments reported here was granted by Lancaster University Research Ethics Committee on 20th December 2012.

The number of participants proving to be sufficient in previous similar studies by the first author, coupled with the fact that the present experiments follow-on very closely from these studies (both conceptually and in terms of experimental design and procedure), indicated that a minimum of 19 participants would be appropriate for each experiment (e.g., this being the number recruited in each of the experiments reported in Walker, 2016b), though other of the relevant studies have been successful with fewer participants (e.g., *N* = 12 in the case of Walker, 2012a). The 60 participants recruited for Experiment 2 reflects our intention to recruit 20 participants to complete each of the conditions based on each of three key sizes, since it was unknown what effect key size would have. Similarly, with regard to Experiment 3, the intention was to examine the between-participant manipulation of articulatory suppression (i.e., absent or present). With this in mind, 20 participants were recruited to complete each of the two conditions created for this purpose.

### Materials

The biggest + heaviest and smallest + lightest objects from the set of nine objects (i.e., objects a & i, respectively, see Fig. 1) served as the two objects that were to be tapped against their assigned contact-sensitive surface to register participants' classification decisions. These two objects are already known to differ significantly in their felt heaviness, and also in their presumed brightness, as judged when they are lifted while being hidden from view (the bigger + heavier object seeming to be the darker of the two) (see Walker et al., 2016).

### Design and procedure

Participants completed four blocks of 36 trials. On each trial a solid circle at one of four brightness levels appeared on the computer screen and participants classified it, as quickly as possible, according to whether it was brighter or darker than the mid-gray background against which it appeared. Their classification decision was communicated by tapping the object held in either their right or left hand against the contact-sensitive surface. Half the participants were instructed to tap with their left hand for brighter circles and with their right hand for dark circles. The other participants were assigned the reverse hand-brightness mapping. A small label was positioned at the bottom of the screen to remind participants of their hand-brightness mapping, and it remained in place for the full duration of the experiment. Which object (heavier + larger or lighter + smaller) was held in the left and right hand was switched between blocks of trials, with the initial object-hand mapping being counterbalanced for each alternative hand-brightness mapping. Within each block of trials every circle was shown nine times, and the order in which the circles were presented was randomized across sets of successive runs of 12 trials, so that within each set every brightness level appeared three times.

The two objects and their contact-sensitive surfaces were covered with a thick black cloth from the outset, and remained hidden from view. Participants were asked to slide their hands under the cloth, grasp one of the two objects in each hand, and hold them approximately 1.5 cm above the appropriate surface. The difference in the size and weight of the two objects was not referred to by the experimenter. Participants were allowed to angle their hands and rest their arms on the table so that they could maintain the same position for a block of trials. They registered their classification decision by tapping the edge of the object being held in the appropriate hand against the contact-sensitive surface beneath. Before each block of trials, participants were allowed to practice tapping the objects and received immediate visual feedback on the computer screen confirming a successful tap. During the trials proper, however, participants did not receive any feedback about the speed or correctness of any of their responses.

During each block of trials, participants listened to a soundtrack, through headphones, comprising randomly timed keyboard strokes. Prior to this, each participant had set the volume of the sound track to a comfortable level, at a time when there would be no incentive to reduce this to a level that would protect performance on a concurrent task. They were not allowed to set the volume at a level that effectively silenced the soundtrack and, in the event, the variation in volume needed to avoid annoyance proved to be relatively modest. After being introduced to the experimental task, it was explained to participants that they did not have to respond to the sounds of the keyboard strokes.

Immediately a response key contacted the surface beneath, the circle disappeared and the response time (RT) was recorded as the time elapsing since the circle appeared. After a 2-s interval, during which the background of the display remained mid-gray, the next circle was presented. Each block of trials took approximately 2 min, and between successive blocks participants moved to another desk to spend 2 min completing a word search task. During this interval, the researcher surreptitiously switched the left-right positioning of the two response keys, with the effect that participants performed each proceeding block of trials with the opposite heaviness-brightness mapping.^5^

## Data analysis

### Analyzing response accuracy

The task instructions emphasised the need to maintain high levels of accuracy, so that the focus would be on response speed as a dependent measure. Levels of accuracy were consistently high in all the experiments being reported here. Simple non-parametric analyses of accuracy focused on confirming the presence, or otherwise, of a heaviness-brightness congruity effect. An alpha level of 0.05 was used for all statistical tests, and only two-tailed *p* values are reported.

### Analyzing response speed

Prior to the statistical analysis of response speed, RTs from error trials, along with RTs identified as being exceptionally fast (< 200 ms) or exceptionally slow (> 2.5 SD above the participant's mean correct RT) were excluded. The remaining correct RTs were subject to reciprocal transformation (i.e., converted to response speed) to improve the normality of the residuals. R (version 3.2.0) (R Core Team, 2012) and lme4 (Bates, Maechler, Bolker, & Walker, 2014) were used to perform linear mixed-effects analyses of the relationship between response speed and the congruence between heaviness and brightness. The intercepts for participants were treated as having a random effect on response speed. For all analyses of this kind, visual inspection of residual Q-Q plots did not reveal any departures from normality that would jeopardize the analysis.

Likelihood-Ratio Tests were used to assess the statistical significance of any impact an individual factor of interest had on response speed, comparing models that differed only in the inclusion of this factor in one of them (and 95% confidence intervals associated with the effect of this factor were calculated using the *Wald* method with the *confint()* function). Alkaike Information Criterion (AIC) values provided a relative estimate of the amount of information not being captured by a model, balancing goodness of fit against the number of parameters the model contains. AICs for alternative models were compared, with lower AIC values indicating a superior model. Finally, unstandardized and standardized estimates of effect size are reported for significant effects of interest, the former estimates in terms of the extent of change in response speed (and mean RT), the latter in terms of two indices. The first index was the percentage of additional variability explained by including heaviness-brightness congruence in the model. Omega-squared (*ω*^2^) as an estimate of variability explained by a model was derived for this purpose (see Xu, 2003), and is seen as equivalent to R^2^ as an index of the correlation between fitted and observed values.^6^ The second index of standardized effect size followed Brysbaert and Stevens' (2018, p. 6) tutorial, and their reference to Westfall, Kenny, and Judd's (2014) recommendations, and was a value equivalent to Cohen's *d*. It is important to point out, however, as Brysbaert and Stevens do, that the variance inherent in RTs is typically large relative to associated effect sizes, so that values for standardized effect sizes are typically very small, being in the region of *d* = 0.1 (with Cohen defining *d* = 0.2 as a small effect size). This needs to be taken into account when reflecting on the values for *d* reported in the present study.

### Results

Incorrect responses were made on 1.9% of trials. A further 3.3% of correct responses were identified as having RT outliers. The resulting mean levels of accuracy and mean correct RTs for *bright* and *dark* classifications made with the *heavy (+big)* and *light (+small)* response keys are summarized in Table 1.

**Table 1.** Experiment 1: Mean correct response times (RTs) (ms) (*SEMs* in parentheses) and *p* (correct) according to between-category brightness and response-key weight (+size), with associated differences in correct RT attributable to key weight

Categorical brightness	Bright	Dark
Response key weight (Size)		
Light (+Small)	797 (13)	798 (13)
	.98	.99
Heavy (+Big)	850 (14)	757 (13)
	.98	.98
Difference (*RT*_*heavy*_ – *RT*_*light*_)		
	53	-41

#### Response accuracy

Overall levels of accuracy on congruent and incongruent trials were 98.4% and 98.0%, respectively. There was not a significant effect of heaviness-brightness congruence on accuracy, Wilcoxon Signed Ranks Test *p* = 0.74.

#### Response speed

The factors entered into the linear mixed-effects analyses of response speed included HEAVINESS (i.e., whether the lighter or heavier response key was being used to register the classification decision), CATEGORICAL BRIGHTNESS (i.e., whether the test stimulus was brighter or darker than the background), WITHIN-CATEGORY BRIGHTNESS (i.e., whether the test stimulus was relatively bright or dark within its category), CONTRAST (i.e., the relative level of brightness contrast between the test stimulus and the background, with black and white stimuli yielding the higher level of contrast, and dark gray and light gray yielding the lower level of contrast), CONGRUENCE (i.e., whether the heaviness of the response key being used was congruent with the level of categorical brightness of the test stimulus), and the CONGRUENCE × CONTRAST interaction.

The average correct response speed was 1.49 responses/s.

**Fig. 2.**
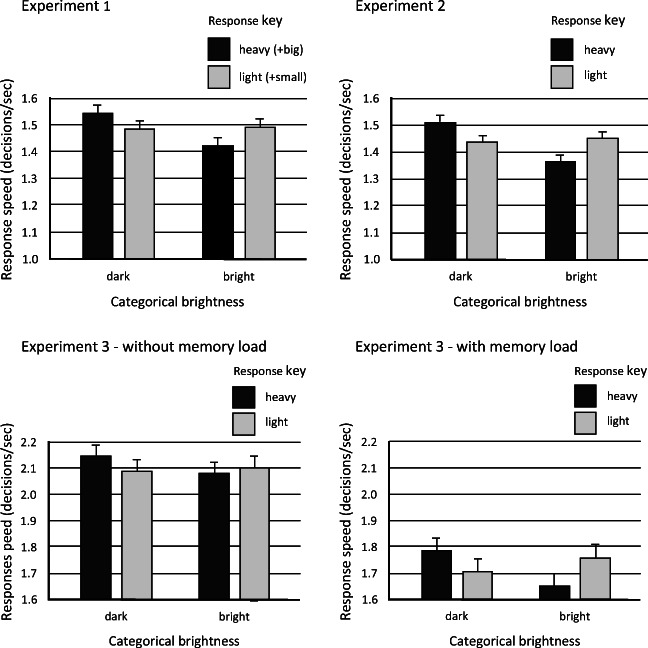
Experiments 1– - 3: Mean correct response speed, with 95% confidence intervals, according to the categorical brightness of the visual test stimulus and the heaviness (and size also in Experiment 1) of the response key needing to be manipulated. In the case of Experiment 3, the results are shown separately for the without and with concurrent memory load task conditions. Note the different range for response speed for Experiment 3

Response speed was faster when the degree of contrast in brightness between the test circle and the background was relatively high (i.e., when a circle was white or black) rather than low (i.e., when a circle was light gray or dark gray), *χ*^2^(1) = 210.64, *p* < .0001, with the higher contrast raising response speed by .21 responses/s (*SE* = .014), CI [.182, .237], reflecting a 130-ms reduction in RT. The categorical brightness of the test stimulus had a significant effect on response speed, *χ*^2^(1) = 20.97, *p* < .0001, with the transition from bright to dark raising response speed by .065 responses/s (*SE* = .014), CI [.037, .093], reflecting a 46 ms reduction in RT. The heaviness of the response key did not have a significant effect on response speed, *χ*^2^(1) = 0.14, *p* = .71. However, heaviness-brightness congruence did have a significant effect on response speed, *χ*^2^(1) = 22.75, *p* < .0001, raising it by .068 responses/s (*SE* = .014), CI [.04, .096], reflecting a 47-ms reduction in RT (see Fig. 2). This effect was linked to a reduction in the AIC value from 5,015 to 4,994 for the null and comparison model, respectively. This is in line with the impact on *ω*^2^ of adding heaviness-brightness congruence to the model, which reveals that an extra 0.4% of the variance was explained, with *ω*^2^ increasing from 29.97% to 30.38%. Cohen's *d* was calculated to be 0.13. The heaviness-brightness congruence effect was not influenced significantly by the degree of brightness contrast between the test circle and the background, *χ*^2^(1) = 0.58, *p* = .44. Finally, when the effect of category brightness on response speed was assessed (using a between-participants' analysis of variance) separately for the light (and small) and heavy (and big) response keys, there was a significant effect for the heavy key, *F*(1,28) = 11.48, *p* = .002, *η*_*p*_^2^ = .29, but not for the lighter weight key, *F*(1,28) = .012, *p* = .91, *η*_*p*_^2^ < .0001.^7^

## Discussion

In the context of the speeded brightness-classification task, Experiment 1 confirms a correspondence between brightness and heaviness and/or between brightness and size. The evidence comes in the form of a correspondence-induced congruence effect (akin to an S-R compatibility effect), wherein participants can classify a visual stimulus for its relative brightness more quickly when the heaviness and/or size of the response key needing to be manipulated to register their classification decision corresponds with the level of brightness of the visual stimulus. For example, participants were quicker to classify a relatively dark visual stimulus (i.e., a stimulus that was darker than the mid-gray background against which it appeared) when this required them to communicate their classification decision by manipulating the heavier + bigger of two response keys. This congruence effect is attributed to the fact that a correspondence between brightness and heaviness/size leads to the heavier + bigger of the two hidden response keys being presumed to be darker than the less heavy + smaller response key. This sense of darkness then feeds into the same conceptual representations of darkness that support the classification decision and, in this way, influence the decision-making process.

As in earlier studies of the correspondence between brightness and size (see Walker & Walker, 2012), the strength of the heaviness-brightness congruence effect proved to be independent of the level of brightness contrast between the visual test stimulus and the mid-gray background against which it appeared. That is, the effect was no different whether it was based on the interaction between heaviness and the two extreme values for brightness (for which brightness contrast was highest), or the interaction between heaviness and the two less extreme values of brightness (for which brightness contrast was lower). This again is taken as evidence that it is simply whether the visual test stimulus is either brighter than the background, or darker than the background, that is entering into correspondence with other cross-sensory features (i.e., what is functionally significant is the dichotomous categorisation of relative brightness that is reflected in the instructions regarding how the stimuli are to be categorised and, on that basis, responded to). Within each of the two brightness categories mapping onto different responses, heaviness-brightness congruence effect was insensitive to any gradations in the degree to which a stimulus is either brighter than the background, or darker than the background. Thus, the two brighter stimuli proved to be indistinguishable from each other in their contribution to the congruence effect, as did the two darker stimuli (see Brunetti, Indraccolo, Del Gatto, & Spence, 2018; Spence, 2019; Walker & Walker, 2016; and Walker, 2016a, for evidence for, and discussion of, the functional significance of the relative, rather than the absolute, coding of stimulus features as they contribute to correspondences).

The success of Experiment 1 instils some optimism regarding the potential value of exploring task situations in which participants communicate their classification decisions by manipulating objects they are holding in their hands, rather than by the more conventional pressing of one or other of two keys on a keyboard.

### Experiment 2: Tapping with response keys contrasting only in weight

Experiment 2 was designed to confirm that the congruence effect observed in Experiment 1 was induced, at least in part, by the contrasting heaviness of the response keys, and not just by their contrasting size. For this reason, it was arranged for participants to register their brightness classification decisions by manipulating two keys which, though differing in weight, were now identical in size. Assuming that the contrasting heaviness of the two response keys in Experiment 1 did contribute significantly to the congruence effect, a similar congruence effect was predicted here. More specifically, it was expected that classification responses would be faster when the heavier object was used to correctly classify visual stimuli as darker, and the lighter weight object was used to correctly classify visual stimuli as brighter, rather than the reverse.

## Method

### Participants

Sixty students completed the study for payment or course credits, though the data for three of them were excluded from the analyses: One participant was removed because of equipment malfunction, and two because they performed at overall levels equivalent to chance (seemingly because they mapped response-key weight, rather than response hand, to contrasting levels of brightness). For the remaining 57 participants (46 females and 11 males), aged between 18 and 48 years (mean age= 19.14 years), 37 had English as their first language. The first languages for the remaining 20 participants were Afrikaans (*n* = 1), Chinese (*n* = 12), Hungarian (*n* = 1), Igbo (*n* = 1), Italian (*n* = 1), Lithuanian (*n* = 1), Malay (*n* = 1), Norwegian (*n* = 1), and Russian (*n* = 1). Eight participants were left-handed by self-report. As stated already, the 60 participants recruited for Experiment 2 reflects our intention to recruit 20 participants for each of the conditions based on the three values for key size, since it was unknown what effect key size would have.

## Materials, design, and procedure

The materials, design, and procedure were essentially the same as in Experiment 1, the principal difference being the choice of objects to be used as response keys. Here, the heaviest and lightest object at each size were paired together to serve as the two alternative response keys for a participant (i.e., object pairs ac, df, & gi, see Fig. 1). Each participant used only one size of response key, with the effect that key size was a between-participant's factor.

## Results

Incorrect responses were made on 1.17% of trials. A further 2.9% of correct trials were identified as having RT outliers. The resulting mean levels of accuracy and mean correct RTs for *bright* and *dark* classifications made with the *heavy* and *light* response keys are summarized in Table 2.

**Table 2.** Experiment 2: Mean correct response times (RTs) (ms) (*SEMs* in parentheses) and *p* (correct) according to between-category brightness and response-key weight, with associated differences in correct RT attributable to key weight

Categorical brightness	Bright	Dark
Response key weight		
Light	828 (11)	847 (11)
	.99	.99
Heavy	890 (11)	778 (11)
	.99	.99
Difference (*RT*_*heavy*_ – *RT*_*light*_)		
	62	-69

### Response accuracy

Overall levels of accuracy on congruent and incongruent trials were 99.3% and 98.6%, respectively, a difference that was significant, Wilcoxon Signed Ranks Test *p* = 0.007.

### Response speed

The factors entered into the linear mixed effects analyses of response speed included HEAVINESS, CATEGORICAL BRIGHTNESS, WITHIN-CATEGORY BRIGHTNESS, CONTRAST, CONGRUENCE, the CONGRUENCE × CONTRAST interaction, SIZE (i.e., whether the paired response keys were large, medium, or small), and the SIZE × CATEGORICAL BRIGHTNESS, SIZE × HEAVINESS, and SIZE × CONGRUENCE interactions.

The average correct response speed was 1.45 responses/s.

**Fig. 3.**
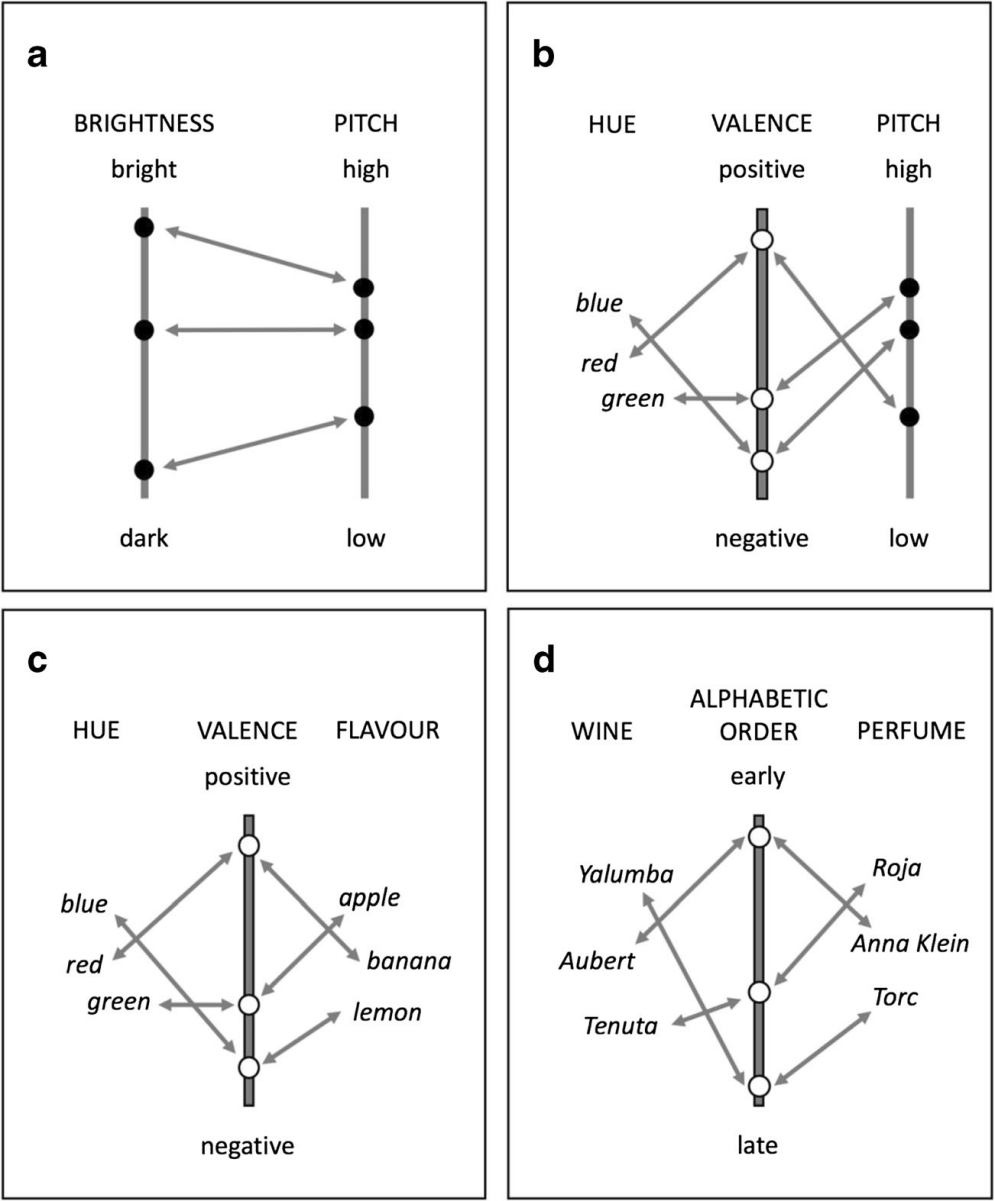
Different ways in which modality-specific features from different domains can be associated with each other, with a potential role for mediating features also being illustrated. (**A**) The notion of cross-sensory correspondences adopted in the present study regards them as reflecting relatively direct associations across aligned feature dimensions (illustrated here with the dimensions of visual brightness and auditory pitch). The individual associations thereby established reflect the context-sensitive and relative mapping of feature values based on them sharing similar ordinal positions within their respective feature sets (i.e., the highest pitch sound in the currently pertinent set of sounds will also be the brightest sound and on this basis will correspond with the brightest visual stimulus in the currently pertinent set). (**B**) Where the different values for a particular feature do not themselves lie along a single dimension, illustrated here with hue, then the mapping of these values onto the values for another feature (here auditory pitch), that do lie on a dimension, can be mediated by mapping both features onto some other dimension, such as valence. In this way, valence can act as a mediator through which the individual values in the two other feature sets become associated. (**C**) A dimension such as valence can serve equally well as a mediator between two sets of features for neither of which do the feature values lie on a dimension (here illustrated with hue and flavour). (**D**) Illustrating how the alphabetic order of a sample of different wines and a sample of different perfumes could, in principle, serve to associate individual wines with individual perfumes

Response speed was faster when the degree of contrast in brightness between the test circle and the background was relatively high, *χ*^2^(1) = 333, *p* < .0001, with the higher contrast raising response speed by .18 responses/s (*SE* = .01), CI [.161, .199], reflecting a 132 ms reduction in RT. The categorical brightness of the test stimulus had a significant effect on response speed, *χ*^2^(1) = 6.07, *p* = .014, with the transition from bright to dark raising response speed by .067 responses/s (*SE* = .027), CI [.014, .12], reflecting a 47-ms reduction in RT. The heaviness of the response key did not have a significant effect on response speed, *χ*^2^(1) = 0.06, *p* = .81. However, heaviness-brightness congruence did have a significant effect on response speed, *χ*^2^(1) = 71.71, *p* < .0001, raising it by .082 responses/s (*SE* = .010), CI [.063, .101], reflecting a 65-ms reduction in RT (see Fig. 3). This effect was linked to a reduction in the AIC value from 9384 to 9314 for the null and comparison model, respectively. This is in line with the impact on *ω*^2^ of adding heaviness-brightness congruence to the model, which reveals that an extra 0.6% of the variance was explained, with *ω*^2^ increasing from 34.85% to 35.45%. Cohen's *d* was calculated to be .158. The heaviness-brightness congruence effect was not influenced significantly by the degree of brightness contrast between the test circle and the background, *χ*^2^(1) = 0.002, *p* = .97. Furthermore, the size of the response keys did not have a significant effect on response speed, *χ*^2^(1) = 2.41, *p* = .12, and did not interact with categorical brightness to yield a between-participants size-brightness congruence effect, *χ*^2^(1) = .021, *p* = .88. However, when the effect of category brightness on response speed was assessed separately for the lighter and heavier of the paired-response keys, there was a significant effect for the heavier key, *F*(1,56.02) = 38.02, *p* < .0001, *η*_*p*_^2^ = .40, but not for the lighter weight key, *F*(1,56.02) = .043, *p* = .51, *η*_*p*_^2^ = .008. Finally, the heaviness-brightness congruence effect was sensitive to the size of the response keys, *χ*^2^(1) = 17.3, *p* < .0001, with each step reduction in key size increasing the congruence effect by .051 responses/s (*SE* = .012), CI [.027, .076]. Separate analyses at each level of key size confirmed the significance of the congruence effect across all three levels. Statistical values relating to the big, medium, and small paired keys, respectively, were: *χ*^2^(1) = 26.32, *p* < .0001, with congruence raising response speed by .06 responses/s (*SE* = .012), CI [.038, .085]; *χ*^2^(1) = 30.90, *p* < .0001, with congruence raising response speed by .09 responses/s (*SE* = .016), CI [.058, .121]; and *χ*^2^(1) = 60.43, *p* < .0001, with congruence raising response speed by .13 responses/s (*SE* = .016), CI [.096, .16], respectively. An explanation of this moderating effect of key size on the congruence effect is offered below.

## Discussion

After arranging for the two response keys to differ only in their weight, the correspondence between heaviness and brightness was observed to induce a congruence effect. In their details, the results of Experiment 2 are remarkably close to those of Experiment 1, suggesting the possibility that it was the relative heaviness of the response keys that induced the congruence effect in Experiment 1.

An important finding observed in Experiment 2 was that the strength of the heaviness-brightness congruence effect appeared once again to be independent of the level of brightness contrast between the visual test stimulus and the mid-gray background against which it appeared. The congruence effect was, therefore, no different whether it reflected the interaction between heaviness and the two extreme values for brightness (for which brightness contrast was highest), or the interaction between heaviness and the two less extreme values of brightness (for which brightness contrast was lower). Thus, within each of the two brightness categories mapping onto alternative responses, the heaviness-brightness congruence effect was again insensitive to any gradations in the degree to which a stimulus was either brighter than the background, or darker than the background. To this extent, the congruence effect appears to be linked to the relative brightness of a visual test stimulus (i.e., relative to the brightness of the background), rather than to its absolute brightness (i.e., relative to some absolute standard, such as black or white).

The significant moderating effect of key size on the heaviness-brightness congruence effect was not anticipated as a primary consideration during the planning of Experiment 2. This effect is interesting, and prompts the question how key size might come to moderate the strength of the congruence effect, especially when the weights of the paired keys remained constant across variations in key size (i.e., the heaviest key in each pair weighed 190 g, and the lightest key 44 g). The answer lies in the distinction between weight and felt heaviness, and the fact that the latter is determined, at least in part, by a combination of size and weight (see Fig. 6 in Walker et al., 2016). The manner in which felt heaviness is determined by a combination of size and weight reflects its sensitivity to an object's material density, a sensitivity that in turn is reflected in the size-weight illusion.

The most common way of characterising the size-weight illusion is to say that as an object gets smaller, while remaining the same weight, it feels to get progressively heavier. However, to the extent that the illusion reflects the influence of an object's material density on felt heaviness, there is an additional way of characterising the illusion, one which links directly to the present experiment. That is, when the weight of one of two identical objects is increased, without changing its other features, the change in its felt heaviness, relative to the heaviness of the unchanging object, will be greater the smaller are the two objects. The reason for this is that, for the same change in weight, the change in material density is greater the smaller is the object.^8^ This leads us to expect that the difference in the felt heaviness of each of the paired keys used in Experiment 2 will increase with each reduction in the size of the keys, that is, it will increase across the object pairings ac, df, and gi, respectively. If one consequence of this is that a difference in felt heaviness will be more likely to be registered during the sensory-perceptual processing of smaller objects, compared to bigger objects, then the heaviness-brightness congruence effect should be more likely to be in evidence (should be stronger overall) for participants using the smaller response keys. This is the nature of the interaction between key size and congruence observed here.

By way of providing evidence in support of this explanation of the moderating effect of key size on the heaviness-brightness congruence effect, additional data were gathered by the authors, albeit after the event, to see if the difference in the felt heaviness of the paired response keys increased as the keys became smaller. Twenty-nine new participants (colleagues and postgraduate students), all blind both to the purpose of the exercise and to the general research area, were presented with all six objects arranged in the same three pairings utilized in Experiment 2. They were asked to lift the two objects in each pair simultaneously, using the thumb and first two fingers of each hand, with a view to judging the difference in their felt heaviness relative to the difference between the other paired objects. They were allowed to lift each of the three pairs of objects, in any order, before declaring their rank ordering regarding the extent of the difference in the felt heaviness of the two paired objects.

The outcome of this modest exercise was clear. Twenty of the participants judged the smallest pair of objects to differ most in their felt heaviness, and the biggest pair of objects to differ least in their felt heaviness, with the difference in felt heaviness of the medium size objects ranking between these two extremes. Notwithstanding the different outcomes for the remaining nine participants, the overall outcome across all participants, as judged using Page's L Test for Linear Ranks (Page, 1963) was significantly as predicted, Page's L = 383, *p* < .001. It appears, therefore, that the degree of difference in the felt heaviness of the paired objects was sensitive to their size in the manner anticipated, with size playing its part via its contribution to determining the material density of each object (see above). Therefore, what might appear to be a direct effect of size on the congruence effect is, instead, an effect mediated by the contribution of size to determining the material density of the objects (see Walker et al., 2016, for additional evidence on this point). Again, therefore, it seems that the cross-sensory features of a hand-held object are determined after the felt heaviness of the object has been influenced by its perceived material density (i.e., after the size-weight illusion has taken effect).

Could brightness be in correspondence directly with material density, rather than felt heaviness? Marks, Hammeal, and Bornstein (1987) consider the possibility that, as a feature, density is not confined to the material density of substances, but instead applies to stimuli in other sensory domains, including simple sounds. They consider that sounds that are higher in loudness and higher in pitch are, *ipso facto,* higher in density, and that this more abstracted notion of density, alongside size (volume), might serve as an overarching feature shared by stimuli encoded in different sensory domains. In which case, density could serve as a basis on which cross-sensory stimuli are judged to correspond. However, given the results from an unpublished study of the similarity scaling of cross-modal relations, Marks et al. reject this notion of a single dimension of density common to, say, hearing and vision, and instead favour a model in which sounds are characterized primarily by their pitch and loudness.

The present study provides relatively direct evidence against material density, rather than size and/or felt heaviness, being the object feature responsible for the presumed heaviness-brightness congruence effect. For example, despite objects A and I having the same material density, they differ in their judged brightness and, because of this, were observed in Experiment 1 to be congruent with contrasting levels of brightness (i.e., the bigger/heavier object, A, proved to be congruent with a darker visual stimulus, and the smaller/lighter object, I, proving to be congruent with a brighter visual stimulus).

### Experiment 3: Closing microswitches on response keys contrasting only in weight, with and without the imposition of a concurrent verbal memory load task

Experiment 3 attempts to replicate the heaviness-brightness congruence effect observed in Experiment 2. At the same time, it begins to examine *how* heaviness contributes to this effect. To do so, the manner in which participants were required to manipulate the objects to communicate their classification decision was changed.

Holding an object in the hand, and tapping it gently against a surface to register a brightness classification decision, ensures the object's heaviness is relevant to the classification response in two ways. First, heaviness has relevance as an enduring feature of the response object, whether or not the object is currently being manipulated to communicate a classification decision. Second, heaviness has relevance as a factor needing to be taken into account when executing the action that will ensure the object is tapped gently against the surface. That is, apart from an object's heaviness being relevant for as long as the object is being held in the hand (i.e., heaviness as an aspect of the object), it has additional relevance for the action that is to be undertaken with the object. Experiment 3 was designed to determine if, after removing heaviness in the second sense, heaviness as an enduring object feature is able to induce a heaviness-brightness congruence effect. To do so, the response keys were altered to reduce markedly, if not eliminate, the relevance of an object's weight to the action needed to communicate a classification decision. This was the rationale for adding a small microswitch to one end of each object. The assumption was that the weight of the object would be largely, if not totally, irrelevant to the act of applying slight pressure with the thumb to close the microswitch by just 2 mm. If a heaviness-brightness congruence effect were still to be observed, then heaviness would begin to appear to have influence in its role as an enduring feature of a response object.

Experiment 3 also assesses the possibility that the heaviness-brightness congruence effect is verbally mediated. Some congruence effects in speeded classification might reflect the fact that cross-sensory feature values in different domains share the same verbal labels, allowing congruence effects to be explained on a purely lexical basis (see Spence, 2011, who allows for lexical explanations of correspondences). An obvious example, in English, is the application of the labels *high* and *low* to contrasting values for both auditory pitch and visuospatial elevation. Such convergence onto the same lexical items provides a potential basis for the congruence effect that is induced by the association between pitch and visuospatial elevation. For example, people might classify the relative spatial elevation of a visual stimulus (e.g., as being *high* rather than *low*) more quickly when it is accompanied by a task-irrelevant sound that is *high* rather than *low* in pitch because both stimuli converge on the same lexical entry, with this entry pointing to, and supporting, the correct classification decision. Of course, the verbal mediation hypothesis requires both the criterial and incidental feature values to be verbally recoded, despite the latter feature being task irrelevant and despite its recoding having the potential to disrupt performance by prompting incorrect classification decisions.

Verbal mediation is of relevance to the heaviness-brightness correspondence, even though the lexical overlap in this case is confined to just one end of the two feature dimensions (i.e., the verbal facilitation of classification responses would be confined to occasions when the response key that is *light* in weight is used to classify a visual stimulus as *bright/light* in color). The more restricted lexical overlap pertaining to this correspondence would not preclude an overall congruence effect being observed, despite the absence of a congruence effect on those occasions where there is no convergence onto the same lexical item, that is, when a *heavy* response key is being used to correctly classify a *darker* visual stimulus.

There is evidence from Experiments 1 and 2, and from Walker and Walker (2012), that speaks against the verbal mediation hypothesis. Though the hypothesis predicts that a heaviness-brightness congruence effect should be in evidence only in relation to the response key that can be labelled as being relatively light in weight, this appears not to be the case. As noted already, and as can be seen from Fig. 1, in Experiments 1 and 2 an effect of categorical brightness on response speed appears only in relation to the heavier of the two response keys. And in Walker and Walker's (2012) study, where the response keys differed in size (and were not lifted), an effect of categorical brightness was confined to the bigger of the two keys. This presents a real challenge to the view that the congruence effect under consideration can be explained solely as arising from overlap in the verbal labelling of feature values. The same finding is, however, inconclusive with regard to other explanations, including the authors' claims regarding the processes underpinning correspondence-induced congruence effects. This is because the restriction of a brightness effect to one key size/weight could simply be an inadvertent consequence of the fact that the interaction between key size/weight and brightness was accompanied by a main effect of brightness (with responses to darker test stimuli being faster than responses to brighter test stimuli). As in all such situations, if one were to adjust the mean values for the four combinations of brightness and key size to remove the main effect of brightness, the result would be a cross-over interaction, with the mean responses to brighter and darker stimuli differing (though in opposite directions) for both levels of key size/heaviness.

Notwithstanding these counter indications, a second condition was introduced to Experiment 3 to assess the verbal mediation hypothesis more directly, that is, by endeavouring to disturb, if not block completely, any task-relevant verbal processes. In previous studies of cross-sensory associations, such as those between the color and taste of different types of fruit (Stevenson & Oaten, 2008), articulatory suppression, in the form of requiring participants to repeatedly utter the word '*the*' alongside completing a different main task, has been used successfully to block verbal recoding of relevant stimulus features (see Reed, 2000; Stevenson & Oaten, 2008; Stevenson, Sundqvist, & Mahmut, 2007). Adopting a slightly different approach to target verbal recoding, participants in this second condition of Experiment 3 were required to complete a concurrent verbal memory load task to disrupt the verbal recoding of both the brightness of the visual test stimulus and the heaviness of the response key needing to be manipulated. Specifically, participants were presented with a spoken sequence of five letters prior to the start of each block of trials in the classification task and had to hold the sequence in mind so that they could recall it in the correct order on completion of the block of trials. Similar concurrent verbal memory load tasks have already been used successfully to assess the involvement of verbal processes in the cross-sensory correspondence between auditory pitch and each of visuo-spatial elevation and visual thinness (Connell, Cai, & Holler, 2013; Dolscheid, Shayan, Majid, & Casasanto, 2013). Where participants perform well in such a concurrent task, it is presumed that sufficient demands have been placed on verbal processes to disrupt the verbal recoding of salient features of the stimuli and response keys in the classification task. In which case, according to the verbal mediation hypothesis, the concurrent task should at least weaken the heaviness-brightness congruence effect.

## Method

### Participants

In line with the intention to have 20 participants complete each condition of the experiment, 40 students (32 females and eight males), aged between 18 and 34 years (mean age = 20.6 years), completed the study for payment or course credits. English was the first language of 29 participants. The first languages of the remaining participants were Bulgarian (*n* = 1), Chinese (*n* = 5), French (*n* = 1), German (*n* = 1), Polish (*n* = 1), Turkish (*n* = 1), and Vietnamese (*n* = 1). Three participants were left handed by self-report. Based on the time they volunteered to participate in the study, 20 students were assigned to each of the *without concurrent memory task condition* and *with concurrent memory task condition.* Data from an additional participant in the latter condition were not included in the analysis because they performed at overall levels equivalent to chance, seemingly because they mapped response-key weight, rather than response hand, to contrasting levels of brightness.

### Materials, design, and procedure

The materials, design, and procedure were the same as for Experiments 1 and 2, but with a different choice of objects to serve as response keys, and with a change in the manner in which these were to be manipulated to register a brightness classification decision. Specifically, in Experiment 3, only the heaviest and lightest medium-sized objects served as the two response keys (i.e., objects D & F, see Fig. 1), with each having a small microswitch attached to the center of one end. Participants held the objects in a manner that allowed them to rest their thumb gently on the microswitch and close it with minimal effort (and minimal movement) to register a classification decision. The concurrent verbal memory load task was added to the situation for the second group of participants.

## Results

### Performance in the concurrent verbal memory task

Recall of a letter sequence was marked as correct only if all the letters were recalled in the correct order. Overall accuracy was 97.5%. On only two occasions was recall incorrect, with one participant reversing the order of two adjacent letters, and another participant replacing two of the letters with letters that had not appeared in the to-be-remembered sequence.

### Performance in the brightness-classification task regardless of the imposition of articulatory suppression

Incorrect responses were made on 1.9% of trials. A further 2.6% of correct responses were identified as having RT outliers. The resulting mean levels of accuracy and mean correct RTs for *bright* and *dark* classifications made with the *heavy* and *light* response keys, separately for the *without* and *with concurrent memory load* conditions, are summarized in Table 3.

**Table 3.** Experiment 3: Mean correct response times (RTs) (ms) (*SEMs* in parentheses) and *p* (correct) according to between-category brightness and response-key weight, with associated differences in correct RT attributable to key weight, separately for those participants who did not undertake a concurrent memory load task, and those who did

Concurrent memory task	Withou		With	
Categorical brightness	Bright	Dark	Bright	Dark
Response-key weight				
Light	528 (8)	528 (8)	687 (13)	675 (13)
	.98	.98	.97	.98
Heavy	542 (8)	518 (8)	711 (13)	655 (13)
	.99	.99	.98	.98
Difference (*RT*_*heavy*_ – *RT*_*light*_)				
	14	-10	24	-20

#### Response accuracy

Overall levels of accuracy on congruent and incongruent trials were 97.8% and 98.4%, respectively, a difference that was not significant, Wilcoxon Signed Ranks Test *p* = 0.21. Adding the concurrent verbal load task did not significantly change overall levels of accuracy, Mann-Whitney U Test *p* = .11.

#### Response speed

The factors entered into the linear mixed effects analyses of response speed included HEAVINESS, CATEGORICAL BRIGHTNESS, WITHIN-CATEGORY BRIGHTNESS, CONTRAST, CONGRUENCE, the CONGRUENCE x CONTRAST interaction, and CONCURRENT MEMORY LOAD (i.e., whether this was in place or not).

Adding the memory load task significantly slowed participants' brightness classification responses, *χ*^2^(1) = 6.95, *p* = .008, reducing response speed by .37 responses/s (*SE* = .14), CI [.109, .641], reflecting a 153 ms increase in RT (see Table 3). Critically, however, in contradiction of the verbal mediation hypothesis, introducing the concurrent task did not significantly reduce the heaviness-brightness congruence effect, *χ*^2^(1) = 2.84, *p* = .09, but instead was linked to a non-significant change of .046 responses/s (*SE* = .027), CI [-.007, .01], equivalent to/reflecting a non-significant increase in the RT congruence effect of 10 ms (i.e., from a difference of 12 ms to a difference of 22 ms). Otherwise, the same pattern of significant and non-significant effects among the main factors was observed regardless of the imposition, or not, of the concurrent task. The presence of the concurrent memory load task failed to interact significantly with the heaviness of the keys, with categorical brightness, or with within-category brightness, *χ*^2^(1) = 1.20, *p* = .27, *χ*^2^(1) < 1, and *χ*^2^(1) < 1, respectively.

### Performance in the brightness-classification task without the concurrent memory load task

#### Response speed

Response speed was faster when the degree of contrast in brightness between the test circle and the background was relatively high, *χ*^2^(1) = 109.53, *p* < .0001, with higher contrast raising response speed by .20 responses/s (*SE* = .02), CI [.161, .234], reflecting 57 ms reduction in RT. The categorical brightness of the test stimulus did not have a significant effect on response speed, *χ*^2^(1) = 2.57, *p* = .11. The heaviness of the response key did not have a significant effect on response speed, *χ*^2^(1) = 0.52, *p* = .47. However, heaviness-brightness congruence did have a significant effect on response speed, *χ*^2^(1) = 4.50, *p* = .034, raising it by .039 responses/s (*SE* = .018), CI [.003, .076], reflecting a 12 ms reduction in RT (see Table 3 and Fig. 1). This effect was linked to a reduction in the AIC value from 3,645 to 3,642 for the null and comparison model, respectively. This is in line with the impact on ω^2^ of adding heaviness-brightness congruence to the model, which reveals that an extra 0.1% of the variance was explained, with ω^2^ increasing from 46.04 to 46.13 %. Cohen's *d* was calculated to be 0.062. The heaviness-brightness congruence effect was not influenced significantly by the degree of brightness contrast between the test circle and the background, *χ*^2^(1) = 0.901, *p* = .34. Finally, when the effect of category brightness on response speed was assessed separately for the light and heavy response keys, the effect for the heavy key fell just short of significance, *F*(1,18.02) = 2.20, *p* = .15, *η*_*p*_^2^ = .11, while the effect for the light-weight key was not significant, *F*(1,18.02) = .08, *p* = .77, *η*_*p*_^2^ = .005.

### *Performance in the speeded classification task with the concurrent memory task*

#### Response speed

Response speed was faster when the contrast in brightness between the test circle and the background was relatively high, *χ*^2^(1) = 226.05, *p* < .0001, with higher contrast raising response speed by .29 responses/s (*SE* = .019), CI [.253, .327], reflecting a 144 ms reduction in RT (see Table 3 and Fig. 2). The categorical brightness of the test stimulus did not have a significant effect on response speed, *χ*^2^(1) = 2.57, *p* = .11. The heaviness of the response key did not have a significant effect on response speed, *χ*^2^(1) = 0.73, *p* = .39. However, heaviness-brightness congruence did have a significant effect on response speed, *χ*^2^(1) = 20.63, *p* < .0001, raising it by .085 responses/s (*SE* = .019), CI [.049, .122], reflecting a 22-ms reduction in RT. This effect was linked to a reduction in the AIC value from 3,672 to 3,653 for the null and comparison model, respectively. This is in line with the impact on ω^2^ of adding heaviness-brightness congruence to the model, which reveals that an extra 0.43% of the variance was explained, with ω^2^ increasing from 45.21to 45.64%. Cohen's *d* was calculated to be 0.136. The heaviness-brightness congruence effect was not influenced significantly by the degree of brightness contrast between the test circle and the background, *χ*^2^(1) = 0.954. When the effect of category brightness on response speed was assessed separately for the light and heavy response keys, there was a significant effect for the heavy key, *F*(1,18.01) = 7.15, *p <* .015, *η*_*p*_^2^ = .28, but not for the light-weight key, *F*(1,18.01) = 1.06, *p* = .31, *η*_*p*_^2^ = .056.

## Discussion

When heaviness as an enduring feature of an object was isolated as the only potential basis for a correspondence-induced congruence effect in the brightness-classification task, such an effect was still observed. Though the strength of the effect was reduced with the change in procedure, it is difficult to say if this reduction came about simply because overall classification speeds were much faster (reflecting the relative ease with which the microswitch on the hidden response key could be pressed, compared to tapping the key on a hidden contact-sensitive surface), or because a component of the congruence effect attributable to heaviness as an aspect of the action needing to be taken had been removed. Therefore, notwithstanding this evidence of a role for heaviness as an enduring feature of a response object, it remains a distinct possibility that heaviness as an aspect of the action needing to be taken with the response object also can provide the basis for a correspondence-induced congruence effect, and did so in Experiments 1 and 2.

The heaviness-brightness congruence effect continued to be observed when participants completed a concurrent verbal memory load task alongside the speeded classification task. Indeed, rather than being reduced by the concurrent task, the congruence effect appeared to be enhanced, despite the memory task being presumed to have interfered with the verbal recoding of both the brightness of the visual test stimulus and the heaviness of the relevant response key. Therefore, the results provide no support for the verbal mediation hypothesis.

Finally, the strength of the heaviness-brightness congruence effect was again insensitive to any variations in the brightness of a visual test stimulus that had no implications for response selection. That is, the effect was sensitive only to variations in brightness that served to determine if the test stimulus was brighter than, or darker than, the mid-gray background against which it appeared. The same theoretical implications regarding the functional significance of the dichotomous coding of relative brightness apply to all three experiments reported here.

## General discussion

The results of the present study confirm that heaviness functions as a feature dimension contributing to cross-sensory correspondences, alongside any separate contribution from size. They also confirm that heaviness contributes in this way when it is a feature of the behavioral response to a stimulus, where it can reveal its influence in the guise of a S-R compatibility effect. More specifically, taking care to dissociate heaviness from size, a heaviness-brightness correspondence was in evidence through its induction of a congruity effect in speeded brightness classification. This effect was observed when heaviness was experienced as the felt heaviness of an object and was a feature associated with the response through which a person communicated their classification decision.

### Heaviness-brightness congruence in brightness classification.

Experiments 2 and 3, along with Experiment 1, provide the most compelling evidence to date for a correspondence between heaviness and brightness, wherein heavy is aligned with dark. The evidence is compelling for three reasons. First, heaviness was instantiated as the felt heaviness of a real object rather than as the presumed heaviness of an item communicated verbally (e.g., where a brighter visual shape is *said* to look lighter in weight than a darker shape). Second, the evidence emerged when influence from the felt heaviness of an object was dissociated from any influence of the object's size, two features that display a very strong link. Third, the evidence comes from a task requiring the speeded classification of visual stimuli in which heaviness (of the response key) is a task-irrelevant feature. Because of its task-irrelevance, it is unlikely that participants deliberately referred to the heaviness of each response key to inform their classification decision. It is also unlikely that participants will have verbally recoded the relative heaviness of the response key needing to be used, which in turn mitigates against the heaviness-brightness congruence effect being verbally mediated. The results of Experiment 3, in which some participants completed a concurrent verbal memory load task, also argue against verbal mediation.

The present study confirms that heaviness contributes to cross-sensory correspondences in its own right, and not simply through its association with size. Together with evidence from other studies (e.g., Hamilton-Fletcher et al., 2018; Walker & Walker, 2012; Walker et al., 2016), size and heaviness are confirmed as being dissociable feature dimensions, each enjoying its own correspondences. This is an important outcome because it helps to validate the identities of the conceptual dimensions involved in cross-sensory correspondences. Without this outcome, it might be claimed that size and heaviness should be collapsed to form a single dimension.

Especially persuasive evidence against collapsing size and heaviness as cross-sensory feature dimensions is provided by Hamilton-Fletcher et al. (2018). As in the present study, their participants were presented with pairs of objects to hold in their hands, where the objects in each pair had been carefully created to contrast in just a single feature. These researchers demonstrate how sighted adults who are blindfolded, and blind adults (including early and late blind adults), are sensitive to the correspondence between auditory pitch and each of heaviness, size, and hardness (with heavier, bigger, and harder all aligning with lower pitch).^9^ By confirming that heaviness and size each enjoy their own correspondence with auditory pitch, and by implication with other cross-sensory features, they help to confirm that the two features should not be collapsed into a single feature. Other cross-sensory dimensions also are candidates for being collapsed, including thickness and size, and thinness and sharpness, and similar attempts to demonstrate their distinctive contributions (or not) are needed.

The presence of a congruity effect in Experiment 3, where participants pressed a microswitch on one of the objects they were holding in their hands, indicates that heaviness as an enduring feature of an object participants are holding, as opposed to a feature needing to be taken into account only when the object is to be manipulated, can provide the basis for a correspondence-induced congruity effect. It still remains a possibility, however, that heaviness as a feature needing to be taken into account only when the object is being manipulated also can induce a correspondence-based congruity effect.

In all three experiments, the heaviness-brightness congruence effect was independent of the level of brightness contrast between the visual test stimulus and the mid-gray background against which it appeared, reaffirming that it is the context-sensitive categorical brightness of a visual stimulus that enters into correspondence, here with heaviness. The same has also been observed, and discussed extensively, in the context of the size-brightness correspondence (see, Walker & Walker, 2012), where it was taken as evidence that it is the relative coding of brightness that contributes most strongly to cross-sensory correspondences. As Walker and Walker (2016) explain, the importance of the relative coding of features is more easily accommodated by the view that the cross-sensory feature dimensions most directly involved in correspondences can be conceptual in nature, and that the congruence effects they induce reflect the crosstalk that takes place among these dimensions, rather than among representations at sensory levels of processing.

### Felt heaviness as a feature of the response by which the classification decision is communicated

In the speeded brightness-classification task used here, heaviness was introduced as a task-irrelevant feature by arranging for the two “response keys” being held in a participant's hands to differ in weight. Though the focus in correspondences research tends to be on features linked to the to-be-classified stimuli, it was reasoned that, because cross-sensory correspondences appear capable of impacting on performance quickly and automatically, and through any sensory domain, a sensory-perceptual feature linked to the means by which a classification decision is communicated (i.e., the response) also will be capable of contributing to a correspondence-based congruity effect. Specifically, it was predicted that a correspondence-based congruence effect would be observed in the form of an S-R compatibility effect, with participants classifying the relative brightness of a visual test stimulus more quickly and/or accurately when this required them to use a key with corresponding heaviness (e.g., the heavier key to classify a darker visual stimulus).

Consistent with cross-sensory correspondences having the potential to have a conceptual basis, rather than a modality-specific sensory basis, they appear able to influence behaviour in many different ways and in many different situations (see Walker, 2016a, for a recent review). Walker and Walker (2012) have already demonstrated that the size of a response key needing to be pressed in a speeded brightness-classification task can induce a congruence effect mediated by the size-brightness correspondence (see an earlier demonstration of this by Walker & Smith, 1985). Not only does this confirm that cross-sensory correspondences extend to embrace proprioception, it also provides an example of a cross-sensory correspondence providing the basis for an S-R compatibility effect (i.e., by determining the level of compatibility between a stimulus and the required response). The present findings extend this to include the heaviness of a “response key” that is held in the hand, with broad and potentially considerable implications for the design of optimum human-machine interfaces.

Equally significant is the encouragement to extend the relevance of correspondences to situations where movements of the body, without the involvement of an object, can more or less successfully communicate simple concepts, such as brightness, according to the degree to which they accord with a relevant cross-sensory correspondence. For example, hand gestures that are more elevated in space, smaller in extent, or quicker, might be especially effective for communicating the relative brightness and sharpness of an object, or the relative pitch of a sound (see Shinohara, Yamauchi, Kawahara, & Tanaka, 2016, for a demonstration close to this).

### Heaviness-brightness correspondence and the concurrent verbal memory load task

Because the heaviness of the response keys was irrelevant to the requirements of the task, at least as specified in the instructions, it is unlikely that participants deliberately referred to the relative heaviness of the response key needing to be manipulated. This in turn mitigates against the heaviness-brightness correspondence, and the congruence effect it induced, being verbally mediated. In this regard, the results of Experiment 3 incorporate another important finding, namely, that the congruity effect induced by the heaviness-brightness correspondence was not reduced when participants completed a concurrent verbal memory load task alongside the brightness-classification task. On the reasonable assumption that the concurrent memory task will have interfered with the verbal recoding of anything other than the to-be-remembered items, this additional observation counters claims that congruity effects based on cross-sensory correspondences in general, and congruity effects based on the heaviness-brightness correspondence in particular, are verbally mediated.

### Where does the heaviness-brightness correspondence originate?

Having revealed a heaviness-brightness correspondence, it now seems important to begin asking where this might originate. Does it, for example, reflect an association between the two features existing in the natural world? As pertinent evidence, of an admittedly anecdotal nature, the first author has examined samples of pebbles collected from different beaches across the UK, along with different types of wood from across the world (provided in Edlin's (1969) book), and found little, or no, association between surface brightness and weight/material density (see Walker, Francis, & Walker, 2010). Despite this, it is worth noting that many materials (e.g., sand, soil, wood, and straw) get both heavier and darker as they get increasingly wet. With this association between the two features, the relative darkness of a material can provide a very valuable clue as to the effort needed to move, or otherwise work with, the material, with this association supporting a broad generalisation that when things become darker they tend also to become heavier.

In addition to the brightness-heaviness association being mediated by wetness, there is also a natural association between the thinness of items, such as leaves, and how lightly colored they appear when illuminated from behind (so that light passes through them). With thicker tending to be heavier, the association between thickness and darkness would mediate an association between heaviness and darkness of color, though this would not be the usual surface darkness linked to the amount of light that is reflected back from the surface of an object. Confirming that there are sufficient instances in the natural world of a heaviness-brightness association to support a general rule linking the two features, and then confirming that this rule is the origin of the correspondence observed in laboratory studies, are exciting challenges awaiting future research.

### The fundamental nature of cross-sensory correspondences

Cross-sensory correspondences, as considered thus far in the present study, involve sets of feature values each from an ordered series (e.g., feature dimensions such as lightness in color, auditory frequency, spatial elevation). Individual feature values across two such sets become associated by virtue of them sharing the same ordinal position within their respective series (see Fig. 3A). As a consequence, knowing how two or more feature values across different sensory domains are linked will constrain the other links established across the same domains, and this is where the principle of transitivity comes into play. For example, if a simple 100-Hz sound corresponds to a spatial elevation (from the ground) of 10°, say, and a sound of 200 Hz corresponds to a spatial elevation of 20°, we can assume that, in general, higher auditory frequency is aligned with higher spatial elevation. On this basis, a 150-Hz sound would be predicted to link with a spatial elevation somewhere between 10 and 20°, and a 400-Hz sound to link with a spatial elevation somewhere in excess of 20°.^10^

Mere associations between individual feature values across domains, by contrast, need not involve ordered sets of feature values, so that individual associations need not be constrained by any other associations linking values across the two domains. We can imagine, for example, that the basic colors (hues) we experience do not belong to a single psychologically ordered set. Consequently, knowing that red is associated with a sound of 200 Hz, say, and green is associated with a sound of 400 Hz, will not allow us to predict the auditory frequency associated with blue. Unlike spatial elevation, hue is not in itself an ordered series able to enter relatively directly into alignment with a set of values from another ordered series.

It is possible, however, that in situations where the feature values in one or both of two domains do not themselves form an ordered series, they can, in effect, come to do so by first mapping onto another dimension that is ordered. Several dimensions have been suggested to serve such a mediating role, including intensity/strength, processing fluency, and valence/pleasantness (cf. Becker, van Rompay, Schifferstein, & Galetzka, 2011; Guetta & Loui, 2017; Spence et al., 2015; Velasco, Hyndman, & Spence, 2018; Velasco, Woods, Petit, Cheok, & Spence, 2016). Taking valence as an example (see Fig. 3B & 3C), and at the risk of oversimplifying matters somewhat, if it were the case that red is highly positively valenced, green moderately valenced, and blue negatively valenced, we would have a basis for systematically linking the colors to sounds with different auditory frequencies on the basis of the valence of the sounds. Notwithstanding having a basis for linking feature values across the two domains, however, there will likely be functional differences between such mediated correspondences and relatively more direct (unmediated) correspondences. For example, only by knowing the valence of a particular color, and the valence of a particular sound, could we anticipate whether they will correspond or not, and only on the basis of valence could transitivity be considered (i.e., green is more positively valenced than blue, red is more positively valenced than green, therefore, red is more positively valenced than blue). Transitivity would not operate on the basis of the values for either color or auditory frequency themselves. Furthermore, were different colors and/or different sounds to share the same value for valence, individual cross-sensory associations would lose something of their uniqueness, with different colors mapping onto the same sound and different sounds mapping onto the same color.

Most stimuli outside the laboratory comprise a mix of feature values, from different sensory feature domains, which very likely incorporate some degree of incoherence among their cross-sensory features. A visual object, for example, might have a pointy shape profile, be relatively large, and have moderate surface brightness, thereby incorporating a significant degree of correspondence-based incongruity (e.g., though a relatively high level of visual pointiness corresponds with a relatively high-pitch sound, a relatively large object corresponds with a relatively low-pitch sound, and a moderate level of surface brightness with a sound of moderate pitch). With such a combination of feature values, cross-sensory correspondences will not be able to link the object as a whole directly to, for example, a simple sound with a specific acoustic frequency, let alone to a complex sound that also incorporates an incoherent (incongruent) mix of feature values. In most everyday situations, therefore, establishing cross-sensory correspondences between complex visual objects and complex stimuli in other domains, such as musical excerpts and flavours, will require them to be mediated correspondences. This suggests that mediated cross-sensory correspondences will be the most frequently encountered type of cross-sensory correspondence.

There is the potential for any number of dimensions to mediate between feature values across different sensory domains, and Schietecat, Lakens, IJsselsteijn, and de Kort (2018a, 2018b) examine how each of the dimensions of affective meaning identified by Osgood, Suci, and Tannenbaum (1957) (i.e., evaluation, potency, and activity) might do so. More specifically, they explore the context-sensitive manner in which a particular affective dimension might become the most salient for this purpose. They consider that the nature of the particular sensory contrast isolated for consideration can determine which dimension of affective meaning becomes most salient as a potential mediator.^11^ For example, a purple color with high saturation and high brightness will link either to the concept of being *aggressive,* or to the contrasting concept of being *calm,* depending whether the other instance of purple against which it is being compared differs from it only in brightness or only in saturation. Where the two purples differ only in brightness, the contrast between them will link most strongly with contrasting values of *evaluation,* whereas, when they differ only in saturation, the contrast between them will link most strongly with contrasting values of *activity*. Thus, whether the saturated bright purple functions as a relatively *negative* stimulus (as if it was *aggressive*), or as a relatively *inactive* stimulus (as if it was *calm*), will depend how it contrasts with the other purple color selected for comparison and, specifically, whether this comparison highlights a contrast in *evaluation* or a contrast in *activity*.

How mediated correspondences differ from the relatively direct cross-sensory correspondences central to the present authors' research remains a moot point. Underlying differences concerning transitivity and the uniqueness of individual correspondences have already been mentioned, and there are likely to be additional such differences discouraging us from regarding both types of correspondence as manifestations of the same underlying processes. As matters stand, the authors would be inclined to recommend that the less direct cross-sensory correspondences are referred to as *mediated cross-sensory correspondences,* especially in contexts where mediation is a pertinent issue. At the same time, however, the authors acknowledge the important task of distinguishing mediated correspondences from other forms of systematic cross-domain associations. For example, it is easy to conceive of alphabetic order serving as a mediator to associate individual products from different categories, such as wines and perfumes (see Fig. 3D). But would such a set of systematic associations qualify as mediated cross-sensory correspondences?

## Footnotes

1 Google Ngram Viewer confirms the appearance of the phrase “thick perfume,” along with “thick odour,” “thick scent,” and “thick stench,” in English language books over the period 1800 to 2008, with “thin perfume,” “thin odour,” “thin scent,” and “thin stench” appearing much less frequently. It also confirms use of the phrases “dark perfume,” “bright perfume,” “heavy perfume,” and “light perfume.” Examples of the use of “thick perfume” include: *“.. and a thick perfume of sweet-fern rises about you”* (Catholic World, 1869); *“.. like a thick perfume wafting around her head”* (Baneberry, 2007); *“A thick perfume tickled Dekker”s nose”* (Bradford, 2015); *“A thick perfume of stale incense hung in the tainted air ..”* (Shearing, 1965); and, *“.. the thick perfume of orange blossoms”* (Silko, 1999).

2 The amount of light provided by a source, and the amount of light coming from a surface, are both often referred to as *brightness*. It is the latter that is of most concern here, and that is referred to as *brightness*.

3 A conceptual basis for correspondences is not incompatible with their appearance in preverbal infants (e.g., Dolscheid, Hunnius, Casasanto, & Majid, 2014; Walker et al., 2010; 2018), chimpanzees (Ludwig, Adachi, & Matsuzawa, 2011), and other non-human animals (e.g., birds, dogs, and frogs all make and exploit the correspondence between pitch of vocalization and body size) (e.g., Morton, 1977). Thus, human infants, chimpanzee infants, and other non-human animals are able to acquire and use abstracted categorical representations of stimuli (e.g., Bomba & Siqueland, 1983; Murai et al., 2005; and, for a comparative review, Zentall, Wasserman, Lazareva, Thompson, & Ratterman, 2008).

4 Brightness, like most of the other features under discussion, is both a modality-specific feature (i.e., visual brightness) and a modality-independent concept (into which visual brightness feeds). The theoretical framework from which predictions are being made focuses on brightness as a modality-independent concept, but in doing so does not deny the reality of brightness as a specifically visual feature too (see Marks, 1978, for a consideration of longstanding claims that brightness can function as a broad, modality-independent concept).

5 The purpose of the word search task was to get participants away from the equipment, thereby allowing the experimenter to switch the left-right positioning of the two keys without this being noticed. Other tasks could have been used, provided they did not have any elements overlapping with the experimental task. The word search task did not have any such overlapping elements, and so was not expected to “contaminate” performance in the main experimental.

6 The R code used to calculate *ω*^2^ is: 1-var(residuals(m))/(var(model.response(model.frame(m)))) *d* was calculated as: (difference between means)/square root(participant variance + residual variance)

7 This result is relevant to the verbal mediation hypothesis and will be discussed as part of the rationale for Experiment 3.

8 Because material density is the weight of an object proportional to its size (as volume), for any specific change in weight there will be a bigger change in material density the smaller the object. Thus, increasing the weight of the bigger, 98 cm^3^ object from 44 g to 190 g increases its material density from 0.45 g/cm^3^ to 1.94 g/cm^3^, whereas doing the same for the smaller, 22 cm^3^ object increases its material density from 2.10 g/cm^3^ to 9.05 g/cm^3^. Whereas in both cases material density is being increased by a factor of 4.35, the change in absolute terms is 1.49 g/cm^3^ in the case of the bigger object and 6.95 g/cm^3^ in the case of the smaller object. The contention here is that the absolute value of an object's material density can contribute to felt heaviness, and that the difference in the felt heaviness of two objects can be sensitive to the absolute difference in their material density, and not just to the two densities expressed as a ratio.

9 This link between hardness and auditory pitch is at odds with at least one form of natural association between these two features. Specifically, it is at odds with evidence that when colliding objects create an impact sound, it is the softness of the objects that serves to selectively dampen the higher acoustic frequencies in the sound. On this point, it is worth noting that Walker and Smith (1984) failed to find a consistent association between these two features when they asked people to rate the hardness-softness of simple sounds differing in pitch. Other research also has revealed hardness to be inconsistently associated with auditory pitch (see Tarte, 1982, for evidence that they are not associated, and Walker et al., 2012, for evidence that they can be associated).

10 A reviewer of the present article kindly pointed out that Parise, Knorre, and Ernst (2014) report evidence indicating that the pitch-elevation correspondence might not be monotonic. At first blush, the graph appearing in Fig. 1B of the Parise et al. article gives the impression that there is a non-monotonic relationship between auditory frequency (pitch) and spatial elevation. However, considered without the extreme two values along the x-axis of this graph, an axis labelled *Band-pass frequency*, the relationship is nicely monotonic, reflecting what is the general understanding in the wider research literature. The extreme two values concern the band-pass frequency ranges of < 0.8 kHz and > 8.0 kHz, that is, frequency ranges that are open-ended at the lower and upper ends, respectively. The result is that the corresponding sounds appear to be qualitatively different from the other sounds (to the present authors at least). This qualitative difference becomes apparent when one listens to the sounds, which Parise et al. kindly make available as *Supporting Information*. Thus, neither of these two extreme sounds appears to have an obviously distinguishable pitch, with the higher band sound seeming not much different from white noise. The exceptional nature of these two sounds, emanating from the open-ended range of extreme frequencies they embrace, appears to link to the reason why Parise et al. excluded them from the statistical analysis used to assess the similarity in the shapes of the frequency-elevation mapping function as determined from their psychophysical task and from the statistics of the stimulus (cf. *p.* 6108 of the Parise et al. article).

11 Schietecat et al. (2018a, 2018b) treat cross-sensory correspondences, as conceived in the present study, as being distinct from the cross-sensory associations mediated by the affective meanings identified by Osgood et al. (1957). Putting aside concerns we might have regarding the manner in which Osgood et al. identify the underlying dimensions of affective meaning, we might question both the logic and the legitimacy of Schietecat et al.'s claim that people attribute positive polarity to one end of each of the three affective dimensions, and that it is the 'more' ends of all these dimensions that are positive (i.e., active and strong are good, whereas passive and weak are bad). Reference to the “more” ends implies that they are all magnitude dimensions, and that 'more' is necessarily positive, which is clearly not the case. For example, a more active 'flu virus is negative rather than positive, just as a stronger odor in a cowshed is likely to be negative. Apart from anything else, by arguing that these dimensions are all aligned with each other in a particular way, Schietecat and colleagues appear to be contradicting the notion that valence, potency, and activity are independent conceptual dimensions, such that, for example, high potency can covary equally with positive and negative valence.

## References

[CR1] Baneberry, Y. (2007). *Bittersweet Nightshade.* Xlibris. *p.* 75.

[CR2] Bates D., Maechler, M., Bolker, B. & Walker, S. (2014). lme4: Linear mixed-effects models using Eigen and S4. R package version 1.1-7, *<URL:* http://CRAN.R-project.org/package=lme4 *>.*

[CR3] Becker L, van Rompay TJL, Schifferstein HNJ, Galetzka M (2011). Tough package, strong taste: The influence of packaging design on taste impressions and product evaluation. Food Quality and Preference.

[CR4] Bomba PC, Siqueland ER (1983). The nature and structure of infant form categories. Journal of Experimental Child Psychology.

[CR5] Bradford, M. (2015). *Button Hill.* Victoria, British Columbia: ORCA Book Publishers. *p.* 24.

[CR6] Brunetti R, Indraccolo A, Del Gatto C, Spence C (2018). Are crossmodal correspondences relative or absolute? Sequential effects on speeded classification. Attention, Perception & Psychophysics.

[CR7] Brysbaert M, Stevens M (2018). Power analysis and effect size in mixed effects models: A tutorial. Journal of Cognition.

[CR8] Catholic World (1869). *Monthly Magazine of General Literature and Science, IX*.

[CR9] Chiou R, Rich AN (2012). Cross-modality correspondence between pitch and spatial location modulates attentional orienting. Perception.

[CR10] Connell L, Cai ZG, Holler J (2013). Do you see what I'm singing? Visuospatial movement biases pitch perception. Brain and Cognition.

[CR11] Deroy O, Crisinel A-S, Spence C (2013). Crossmodal correspondences between odors and contingent features: Odors, musical notes, and geometric shapes. Psychonomic Bulletin and Review.

[CR12] Dolscheid S, Hunnius S, Casasanto D, Majid A (2014). Prelinguistic infants are sensitive to space-pitch associations found across cultures. Psychological Science.

[CR13] Dolscheid S, Shayan S, Majid A, Casasanto D (2013). The thickness of musical pitch: Psychophysical evidence for linguistic relativity. Psychological Science.

[CR14] Edlin HL (1969). *What wood is that? A manual of wood identification*.

[CR15] Evans K, Treisman A (2010). Natural cross-modal mappings between visual and auditory features. Journal of Vision.

[CR16] Gallace A, Spence C (2006). Multisensory synesthetic interactions in the speeded classification of visual size. Perception & Psychophysics.

[CR17] Guetta, R., & Loui, P. (2017). When music is salty: The crossmodal associations between sound and taste. *PLoS One, 12,* e0173366. doi: 10.1371/journal.pone.0173366 10.1371/journal.pone.0173366 PMC5371278 28355227

[CR18] Gundlach C, Macoubray C (1931). The effect of color on apparent size. American Journal of Psychology.

[CR19] Hamilton-Fletcher G, Pisanski K, Reby D, Stefanczyk M, Ward J, Sorokowska A (2018). The role of visual experience in the emergence of cross-modal correspondences. Cognition.

[CR20] Karwoski TF, Odbert HS, Osgood CE (1942). Studies in synesthetic thinking: II. The role of form in visual responses to music. The Journal of General Psychology.

[CR21] Lidji P, Kolinsky R, Lochy A, Morais J (2007). Spatial associations for musical stimuli: A piano in the head?. Journal of Experimental Psychology: Human Perception and Performance.

[CR22] Ludwig VU, Adachi I, Matsuzawa T (2011). Visuoauditory mappings between high luminance and high pitch are shared by chimpanzees *Pan troglodytes* and humans. Proceedings of the National Academy of Sciences of the United States of America.

[CR23] Ludwig VU, Simner J (2013). What color does that *feel*? Tactile-visual mapping and the development of cross-modality. Cortex.

[CR24] Marks LE (1978). *The Unity of the Senses: Interrelations among the modalities*.

[CR25] Marks LE (1987). On cross-modal similarity: Auditory–visual interactions in speeded discrimination. Journal of Experimental Psychology: Human Perception and Performance.

[CR26] Marks LE, Hammeal RJ, Bornstein MH (1987). Perceiving similarity and comprehending metaphor. Monographs of the Society for Research in Child Development.

[CR27] Martino G, Marks LE (1999). Perceptual and linguistic interactions in speeded classification: Tests of the semantic coding hypothesis. Perception.

[CR28] Martino G, Marks LE (2001). Synesthesia: Strong and weak. Current Directions in Psychological Science.

[CR29] Melara RD (1989). Dimensional interaction between color and pitch. Journal of Experimental Psychology: Human Perception and Performance.

[CR30] Melara RD, Marks LE (1990). Processes underlying dimensional interactions: Correspondences between linguistic and non-linguistic dimensions. Memory and Cognition.

[CR31] Morton ES (1977). On the occurrence and significance of motivation-structural rules in some bird and mammal sounds. American Naturalist.

[CR32] Murai C, Kosugi D, Tomonaga M, Tanaka M, Matsuzawa T, Itakura S (2005). Can champanzee infants (*Pan troglodytes*) form categorical representations in the same manner as human infants (*Homo sapiens*)?. Developmental Science.

[CR33] Nava E, Grassi M, Turati C (2016). Audio-visual, visuo-tactile and audio-tactile correspondences in preschoolers. Multisensory Research.

[CR34] Occelli V, Spence C, Zampini M (2009). Compatibility effects between sound frequency and tactile elevation. NeuroReport.

[CR35] Osgood CE, Suci GJ, Tannenbaum PH (1957). *The Measurement of Meaning*.

[CR36] Page EB (1963). Ordered hypotheses for multiple treatments: A significance test for linear ranks. Journal of the American Statistical Association.

[CR37] Parise CV, Knorre K, Ernst MO (2014). Natural auditory scene statistics shapes human spatial hearing. Proceedings of the National Academy of Sciences of the United States of America.

[CR38] R Core Team (2012). R: A language and environment for statistical computing.

[CR39] Reed P (2000). Serial position effects in recognition memory for odors. Journal of Experimental Psychology. Learning, Memory, and Cognition.

[CR40] Ro T, Hsu J, Yasar NE, Elmore LC, Beauchamp MS (2009). Sound enhances touch perception. Experimental Brain Research.

[CR41] Robinson EJ (1954). The influence of photometric brightness on judgements of size. American Journal of Psychology.

[CR42] Rusconi E, Kwan B, Giordano BL, Umilta C, Butterworth B (2006). Spatial representation of pitch height: the SMARC effect. Cognition.

[CR43] Schietecat AC, Lakens D, IJsselsteijn WA, de Kort YAW (2018). Predicting context-dependent cross-modal associations with dimension-specific polarity attributions. Part 1: Brightness and aggression. Collabra: Psychology.

[CR44] Schietecat AC, Lakens D, IJsselsteijn WA, de Kort YAW (2018). Predicting context-dependent cross-modal associations with dimension-specific polarity attributions. Part 2: Red and valence. Collabra: Psychology.

[CR45] Shearing, J. (1965). *Aunt Beardie.* New York: Berkley Publishing Corporation. *p.* 89.

[CR46] Shinohara, K., Yamauchi, N., Kawahara, S., & Tanaka, H. (2016). *Takete* and *Maluma* in action: A cross-modal relationship between gestures and sounds. *PLoS One, 11,* e0163525. doi: 10.1371/journal.pone.0163525 10.1371/journal.pone.0163525 PMC5040269 27682989

[CR47] Silko, L. M. (1999). *Gardens in the Dunes.* New York: Simon & Schuster. *p.* 83.

[CR48] Spence C (2011). Crossmodal correspondences: A tutorial review. Attention, Perception, & Psychophysics.

[CR49] Spence C (2019). On the relative nature of (pitch-based) crossmodal correspondences. Multisensory Research.

[CR50] Spence, C., Wan, X., Woods, A., Velasco, C., Deng, J., Youssef, J., & Deroy, O. (2015). On tasty colors and colorful tastes? Assessing, explaining, and utilizing crossmodal correspondences between colors and basic tastes. *Flavour, 4:23* doi 10.1186/s13411-015-0033-1

[CR51] Stevenson RJ, Oaten M (2008). The effect of appropriate and inappropriate stimulus color on odor discrimination. Perception & Psychophysics.

[CR52] Stevenson RJ, Sundqvist N, Mahmut M (2007). Age-related changes in discrimination of unfamiliar odors. Perception & Psychophysics.

[CR53] Tarte RD (1982). The relationship between monosyllables and pure tones: An investigation of phonetic symbolism. Journal of Verbal Learning and Verbal Behavior.

[CR54] Velasco C, Hyndman S, Spence C (2018). The role of typeface curvilinearity on taste expectations and perception. International Journal of Gastronomy and Food Science.

[CR55] Velasco C, Woods AT, Marks LE, Cheok AD, Spence C (2016). The semantic basis of taste-shape associations. PeerJ.

[CR56] Velasco C, Woods AT, Petit O, Cheok AD, Spence C (2016). Crossmodal correspondences between taste and shape, and their implications for product packaging: A review. Food Quality and Preference.

[CR57] Walker P (2012). Cross-sensory correspondences and crosstalk between dimensions of connotative meaning: Visual angularity is hard, high-pitched, and bright. Attention, Perception, & Psychophysics.

[CR58] Walker P (2012). Cross-sensory correspondences and naïve conceptions of natural phenomena. Perception.

[CR59] Walker P (2016). Cross-sensory correspondences: A theoretical framework and their relevance to music. Psychomusicology: Music, Mind and Brain.

[CR60] Walker P (2016). Cross-sensory correspondences and symbolism in spoken and written language. Journal of Experimental Psychology. Learning, Memory, and Cognition.

[CR61] Walker P, Bremner JG, Lunghi M, Dolscheid S, Dalla Barba B, Simion F (2018). Newborns are sensitive to the correspondence between auditory pitch and visuospatial elevation. Developmental Psychobiology.

[CR62] Walker P, Bremner JG, Mason U, Spring J, Mattock K, Slater A, Johnson SP (2010). Preverbal infants’ sensitivity to synaesthetic cross-modality correspondences. Psychological Science.

[CR63] Walker P, Francis BJ, Walker L (2010). The brightness-weight illusion: Darker objects look heavier but feel lighter. Experimental Psychology.

[CR64] Walker P, Scallon G, Francis BJ (2016). Cross-sensory correspondences: Heaviness is dark and low-pitched. Perception.

[CR65] Walker P, Smith S (1984). Stroop interference based on the synaesthetic qualities of auditory pitch. Perception.

[CR66] Walker P, Smith S (1985). Stroop interference based on the multimodal correlates of haptic size and auditory pitch. Perception.

[CR67] Walker P, Walker L (2012). Size-brightness correspondence: Crosstalk and congruity among dimensions of connotative meaning. Attention, Perception, & Psychophysics.

[CR68] Walker L, Walker P (2016). Cross-sensory mapping of feature values in the size-brightness correspondence can be more relative than absolute. Journal of Experimental Psychology: Human Perception and Performance.

[CR69] Walker L, Walker P, Francis BJ (2012). A common scheme for cross-sensory correspondences across stimulus domains. Perception.

[CR70] Walker P, Walker L, Francis BJ (2015). The size-brightness correspondence: Evidence for crosstalk among aligned conceptual feature dimensions. Attention, Perception & Psychophysics.

[CR71] Wallis WA (1935). The influence of color on apparent size. Journal of General Psychology.

[CR72] Westfall J, Kenny DA, Judd CM (2014). Statistical power and optimal design in experiments in which samples of participants respond to samples of stimuli. Journal of Experimental Psychology: General.

[CR73] Xu R (2003). Measuring explained variation in linear mixed effect models. Statistics in Medicine.

[CR74] Zentall TR, Wasserman EA, Lazareva OF, Thompson RKR, Ratterman MJ (2008). Concept learning in animals. Comparative Cognition & Behavior Reviews.

